# Molecular insights into atmospheric methane-oxidizing USCγ from desert grassland soil based on metagenome-assembled genome analysis

**DOI:** 10.1093/ismeco/ycag151

**Published:** 2026-06-03

**Authors:** Yufang Wang, Yuanfeng Cai, Zechen Peng, Fujiang Hou, Zhongjun Jia

**Affiliations:** State Key Laboratory of Soil and Sustainable Agriculture, Institute of Soil Science, Chinese Academy of Sciences, Nanjing, Jiangsu Province 211135, P.R. China; Key Laboratory of Microbial Resources Exploitation and Application of Gansu Province, Institute of Biology, Gansu Academy of Sciences, Lanzhou, Gansu Province 730000, P.R. China; State Key Laboratory of Herbage Improvement and Grassland Agro-Ecosystems, College of Pastoral Agriculture Science and Technology, Lanzhou University, Lanzhou, Gansu Province 730020, P.R. China; State Key Laboratory of Soil and Sustainable Agriculture, Institute of Soil Science, Chinese Academy of Sciences, Nanjing, Jiangsu Province 211135, P.R. China; State Key Laboratory of Herbage Improvement and Grassland Agro-Ecosystems, College of Pastoral Agriculture Science and Technology, Lanzhou University, Lanzhou, Gansu Province 730020, P.R. China; State Key Laboratory of Herbage Improvement and Grassland Agro-Ecosystems, College of Pastoral Agriculture Science and Technology, Lanzhou University, Lanzhou, Gansu Province 730020, P.R. China; State Key Laboratory of Soil and Sustainable Agriculture, Institute of Soil Science, Chinese Academy of Sciences, Nanjing, Jiangsu Province 211135, P.R. China; Northeast Institute of Geography and Agroecology, Chinese Academy of Science, Changchun, Jinlin Province 130102 P.R. China

**Keywords:** USCγdesert grassland, metagenome-assembled genome, *pmoA*

## Abstract

Upland Soil Cluster Gamma (USCγ) is a key high-affinity aerobic methanotroph driving atmospheric methane oxidation in grassland soils; however, it has never been obtained in pure culture, and its metabolic processes remain largely unknown. Here, we reconstructed a USCγ metagenome-assembled genome (MAG) containing the complete *pmoA* gene from desert grassland soil in northwestern China, designated USC_AKS. At the site, USCγ accounted for 9.83% of the microbial community in the 10–20 cm layer. BLASTn of its 16S rRNA gene against the NCBI database (excluding uncultured/environmental sequences) showed 93.03% similarity to the non-methanotroph *Thioalkalivibrio sulfidiphilus* HL-EbGr7 (order Chromatiales). The closest match among named species was an uncultured bacterium (JN672117) at 97.86% similarity. Its *pmoA* shares 96.18% similarity with the original USCγ-defining sequence. Phylogenomic analysis placed USC_AKS and seven other USCγ MAGs into a monophyletic group of three subclades, distantly related to culturable Type I methanotrophs. Their genomic average nucleotide identity values are all below 95%, confirming eight distinct species. Like other USCγ MAGs, USC_AKS encodes a complete *pmoCAB* operon, an *XoxF*-type methanol dehydrogenase, and enzymes for formaldehyde oxidation to CO_2_. However, it lacks key ribulose monophosphate (RuMP) cycle genes encoding 3-hexulose-6-phosphate synthase (*hps*) and 6-phospho-3-hexulose isomerase (*phi*). The serine cycle also appears incomplete, as these MAGs lack *hpr*, the gene encoding hydroxypyruvate reductase. Moreover, none encode Rubisco, ruling out the Calvin–Benson–Bassham CO_2_-fixation pathway. Consequently, the metabolic characteristics of USCγ—particularly its carbon assimilation pathway—remain enigmatic, and obtaining pure cultures or enriched consortia is likely the only route to resolving this mystery.

## Introduction

Soil microbial activity constitutes the sole biological process capable of oxidizing atmospheric methane at trace levels (~1.946 ppmv) [1]. Among aerobic methanotrophs, the uncultivated Upland Soil Cluster Alpha (USCα) and Upland Soil Cluster Gamma (USCγ) are considered the primary drivers of atmospheric methane oxidation [2]. USCα is frequently detected in acidic to neutral environments [3], whereas USCγ prevails in alkaline grassland soils, such as those in the Loess Plateau [4], Inner Mongolia [5], and Xinjiang, China [6, 7]. To date, no USCγ strain or enrichment culture has been obtained, and information on its physiological and metabolic characteristics remains very limited.

Aerobic methanotrophs are traditionally classified into Gammaproteobacterial (Type I) and Alphaproteobacterial (Type II) based on their membrane architecture and carbon assimilation pathways [1, 8]. The core carbon assimilation pathway of Type I methanotrophs is the ribulose monophosphate (RuMP) pathway, whereas the characteristic pathway of Type II methanotrophs is the serine cycle. USCα belongs to Type IIb, while USCγ is classified as Type Id [8]. However, it should be noted that genes encoding serine cycle enzymes can occur in both Type I and Type II methanotrophs, and a complete set of the entire cycle is not always present [9].

Metagenome-assembled genome (MAG) analysis represents an important approach for investigating the metabolism of unexplored lineages and certain novel methanotrophs [10]. Existing MAG analyses of various methanotrophs have revealed new functional traits and metabolic details [11, 12]. Compared with USCγ, studies on USCα (including the RA14, JR1/Cluster 5, and MHP clades) have been relatively comprehensive [13, 14]. For example, the MAG of the USCα taxon *Candidatus* Methyloaffinis lahnbergensis, derived from forest soils, was found to harbor numerous genes for the synthesis of extracellular polysaccharides associated with biofilm formation [13].

To date, only a few MAGs have been recovered for the USCγ clade. In 2017, the first high-affinity methanotroph genome from Antarctic mineral permafrost was assembled and designated USCγ_Taylor. The authors reported that USCγ_Taylor, which belongs to the Type I methanotrophs (Gammaproteobacteria), contained an almost complete serine pathway, which is typically associated with Type II methanotrophs, rather than the ribulose monophosphate (RuMP) pathway commonly found in Type I methanotrophs. Furthermore, its 488 nt-long 16S rRNA gene shared 99% identity with uncultured bacteria (JQ684308, HM445440, and DQ823229) and 94% identity with *Thioalkalivibrio* (NR_074692) and *Ectothiorhodospira* (NR_125567) of the order Chromatiales, a group phylogenetically closely related to *Methylococcales* but containing no known methanotrophs [15]. In 2021, MGR_bin175 (~93% complete, 4.42% contaminated) from Antarctica’s Mackay Glacier region was reported to carry USCγ *pmoA* genes, although these sequences were not analyzed in depth [16]. More recently, USCγ MAGs have been reported from Lehman Cave wb1-P19 in Great Basin National Park, Nevada, USA, and from four aerated limestone and basalt caves in Australia. The Lehman Cave wb-P19 MAG encodes particulate methane monooxygenase (*pmoCAB*), enzymes involved in methanol, formaldehyde, and formate oxidation, and a complete serine cycle, but lacks Rubisco and known CO_2_ fixation pathways [17]. The four MAGs from Australian caves also lack key genes for the serine cycle, RuMP pathway, and Calvin–Benson–Bassham (CBB) cycle, leaving their carbon fixation mechanisms unresolved [18]. Importantly, these findings remain far from adequate to provide a comprehensive depiction of the physiology and metabolism of USCγ.

Here, we obtained a USCγ MAG (USC_AKS) from a temperate desert soil in northwestern China through metagenomic sequencing and assembly. By reconstructing its central metabolism, placing it in a phylogenetic context, and systematically comparing it with seven published MAGs, we provide new data that contribute to a comprehensive view of the USCγ metabolic network.

## Materials and methods

### Soil sampling

The soil samples used in this study were collected in September 2019 from a temperate desert in Aksai Kazakh Autonomous County, Gansu Province, China (39°8′N, 94°6′E), at an elevation of 3370 m above sea level (a.s.l.). The site has a mean annual temperature of 1–2°C and a mean annual precipitation of 20–80 mm. The dominant grassland species at this location include *Salsola laricifolia, Atraphaxis pungens*, and *Juncus bufonius*. After the sampling points were selected, four areas of 10 m × 10 m plots were designated as biological replicates (R1 to R4), with a minimum distance of 100 m between plots. In each plot, four soil cores were randomly collected at 10 cm intervals to a depth of 40 cm. The four soil layers obtained at each depth were then combined to form a composite sample. Fresh soil samples were transported to the laboratory in a refrigerated container and then sieved through a 2 mm mesh to remove plant roots, debris, and gravel. The samples were subsequently stored at −20°C for DNA analysis and metagenomic sequencing.

Soil particle composition was analyzed using a laser particle size analyzer (LS13320, Beckman Coulter, Inc.), after sieving (2 mm) 5 g of soil that had been air-dried at room temperature for seven days. Soil moisture, pH, total carbon (TC), and total nitrogen (TN) (g kg^−1^), as well as ammonium (NH_4_^+^–N) and nitrate (NO_3_^−^–N) (mg kg^−1^) nitrogen, were measured as previously described by Wang *et al.* [4]. The results of soil particle composition and soil physicochemical properties are shown in Table 1.

**Table 1:** Physicochemical properties of AKS soils at different depths.

	Depths			
	0–10 cm	10–20 cm	20–30 cm	30–40 cm
Particle size composition				
<5 μm	25.68 ± 0.54 a	24.50 ± 1.07 a	22.78 ± 1.04 a	24.50 ± 1.99 a
5–10 μm	9.40 ± 0.11 a	9.50 ± 0.36 a	9.58 ± 0.50 a	10.03 ± 0.22 a
10–50 μm	32.68 ± 0.62 b	34.33 ± 0.76 ab	35.55 ± 0.67 a	36.45 ± 0.71 a
50–100 μm	20.58 ± 0.25 a	20.18 ± 1.11 a	20.05 ± 1.11 a	19.53 ± 0.97 a
100–500 μm	11.45 ± 0.43 ab	11.30 ± 0.61 ab	11.65 ± 0.92 a	9.46 ± 0.51 b
500–1500 μm	0.23 ± 0.11 ab	0.20 ± 0.04 ab	0.40 ± 0.17 a	0.04 ± 0.02 b
pH	8.73 ± 0.08 a	8.76 ± 0.09 a	8.79 ± 0.09 a	8.84 ± 0.08 a
Soil Moisture, %	9.79 ± 0.75 b	12.94 ± 0.88 a	14.32 ± 0.47 a	14.46 ± 0.55 a
NO_3_^—^N, mg kg^−1^	2.51 ± 0.49 a	1.93 ± 0.24 ab	1.48 ± 0.07 b	1.52 ± 0.06 b
NH_4_^+^–N, mg kg^−1^	8.48 ± 1.64 a	7.90 ± 1.53 a	7.01 ± 0.42 a	7.27 ± 0.46 a
Total nitrogen, g kg^−1^	0.59 ± 0.03 a	0.50 ± 0.06 a	0.34 ± 0.04 b	0.36 ± 0.01 b
Total carbon, g kg^−1^	25.95 ± 0.30 a	25.58 ± 1.38 a	24.25 ± 1.03 a	24.68 ± 0.90 a

Note: Data followed by different characters in a row are significant differences (*P* < 0.05). Data followed by character ab means no significant differences (*P* > 0.05) with other data in the same row.

Total genomic DNA was extracted from AKS soil samples using the Mag-Bind® Soil DNA Kit (Omega Bio-tek, Norcross, GA, USA) according to the manufacturer's instructions. DNA concentration and purity were determined using a TBS-380 and a NanoDrop 2000, respectively. DNA quality was checked on a 1.20% agarose gel, and the samples were then stored at −20°C for amplicon sequencing.

### Real-time quantitative PCR and MiSeq sequencing of the 16S rRNA and *pmoA* genes

For the 16S rRNA gene, the primers 515F/907R [19] were used to determine bacterial abundance via real-time quantitative PCR (qPCR). The details of the qPCR procedure have been described previously [4]. The amplification efficiency for the 16S rRNA gene was 92.60%, with an *R*^2^ value of 0.99. Additionally, a 7-bp sample-specific adaptor sequence was attached to the 5′ end of 515F primer for Illumina MiSeq sequencing to simultaneously assess the community composition of methanotrophs and their relative abundance within the total bacterial population. For the *pmoA* gene, the primers A189f/A682r [20] were used to determine methanotroph abundance using qPCR. The amplification efficiency for the *pmoA* gene was 90.30%, with an *R*^2^ value of 0.99. The primer pairs A189f/A682r and A189f/mb661r/A650 [21] were used for semi-nested PCR amplification to characterize the methanotroph community composition in the soil as described previously [22].

All sequencing amplicons were verified on 1.20% (w/v) agarose gels stained with GoldView. Bands of the expected size (~390 bp for the 16S rRNA gene and 510 bp for the *pmoA* gene) were excised and purified using the MiniBEST DNA Fragment Purification Kit ver. 3.0 (TaKaRa Bio Inc., Kusatsu, Japan). The purified PCR products were quantified using a NanoDrop ND-2000 spectrophotometer and mixed at an equimolar ratio. A library was constructed, and sequencing was performed on the Illumina MiSeq platform using the MiSeq kit v3 for 2 × 300 bp paired-end sequencing (Majorbio Bio-Pharm Technology Co., Ltd., Shanghai, China).

Quality filtering, denoising, paired-end read merging, and chimera removal were performed on the raw FASTQ data of the 16S rRNA and *pmoA* genes using Mothur software version 1.41.3 [23] according to the online instructions for the relevant commands (https://mothur.org/wiki/tags/#commands). Reads with lengths of 370–380 nt were selected for the 16S rRNA gene, and those with lengths of 470–471 nt were selected for the *pmoA* gene. Chimeras were filtered out using the “chimera.vsearch” and “remove.seqs” commands. Finally, high-quality sequences were classified directly using the “classify.seqs” command with a Naïve Bayes classifier at a confidence threshold of 80% [24]. For the 16S rRNA gene, all sequences were clustered into operational taxonomic units (OTUs) at 97% sequence similarity. The representative OTU sequences were further identified via BLASTn search against the modified RDP database [25]. *pmoA* sequences were clustered on their deduced amino acid sequences by FunGene Pipeline with a distance cutoff of 0.07 [26] and classified using a method previously described [22].

### Metagenomic sequencing, assembly, binning

DNA from the AKS-R1 and AKS-R2 soil samples from the 10–20 cm layer was fragmented to an average size of ~400 bp using a Covaris M220 (Gene Company Limited, China) for paired-end library construction. The paired-end library was constructed using the NEXTFLEX Rapid DNA-Seq Kit (Bioo Scientific, Austin, TX, USA). Adapters containing the full complement of sequencing primer hybridization sites were ligated to the blunt ends of the fragments. Paired-end sequencing was performed on the Illumina NovaSeq platform (Illumina Inc., San Diego, CA, USA) at Majorbio Bio-Pharm Technology Co., Ltd. (Shanghai, China) using the NovaSeq 6000 S4 Reagent Kit v1.5 (300 cycles), according to the manufacturer’s instructions.

For each soil sample, 30 Gb of raw paired-end reads were generated and uploaded to the KBase platform for processing, which included read merging, assembly, and binning [27]. Adapters and low-quality bases were removed using Trimmomatic v0.36 on the KBase platform with default parameters (ILLUMINACLIP:TruSeq3-PE.fa:2:15:4:1:true, SLIDINGWINDOW:4:15, LEADING:3, TRAILING:3, MINLEN:36) to obtain high-quality clean data [28]. Reads were assembled concurrently using metaSPAdes v3.13.0 [29] and MEGAHIT v1.2.9 [30] with default settings. Contigs ≥1.50 kb were binned using MetaBAT v2.12.1 [31] and MaxBin2 v2.2.4 [32], and the resulting bins were refined and consolidated with DAS Tool to select methanotroph MAGs [33]. Completeness and contamination were evaluated with CheckM v1.0.17 [34], and taxonomic assignment was performed with GTDB-Tk v1.7.0 [35]. Protein-coding genes were predicted using both Prokka v1.14.5 [36] and Prodigal [37]. Functional annotation was carried out with BlastKOALA against the Kyoto Encyclopedia of Genes and Genomes (KEGG) database [38].

### Phylogenetic analysis

To infer phylogenetic affinities, the full-length *pmoA* and 16S rRNA genes were extracted from the MAG USC_AKS and combined with dominant USCγ OTUs obtained via amplicon sequencing in this study. For the *pmoA* gene tree, reference sequences included those from representative strains of Type I (e.g. *Methyloculum, Methylosarcina, Methylobacter*) and Type II (e.g. *Methylocapsa, Methylosinus, Methylocystis*) methanotrophs, as well as those from other USCγ MAGs (e.g. Lehman Cave wb1-P19, Frasassi Cave wb1-P19, USCγ_Taylor, MGR_bin175, SD8037_metabat2.bin.6, SD8020_metabat2.bin.3, and H1-B1_maxbin2.bin.5.sub). Additionally, several USCγ-related environmental sequences (uncultured bacterium clone sequences) available in GenBank, such as LHN-44 (MN566549), WR412 (MF999971), and XxxM0454 (KY606372), were also included. For the 16S rRNA gene tree, reference sequences included those from representative strains of Type I (e.g. *Methylomonas, Methylomagnum*) and Type II (e.g. *Methylocapsa, Methylocystis, Methylosinus*) methanotrophs, as well as one from MGR_bin175. Additionally, several USCγ-related environmental sequences (uncultured bacterium clone sequences) available in GenBank, such as B8-283 (KF494664), ORCA-17N118 (DQ823220), and GP27677hO1 (HM445440), were also included. Neighbor-joining trees were constructed in MEGA 7.0 using the Kimura 2-parameter (K2P) model for 16S rRNA and *pmoA* gene sequences with 1000 bootstrap replicates, pairwise deletion of gaps, and uniform rates among sites. Phylogenetic analysis of the *XoxF* protein sequence encoded in the USCγ MAGs was performed using IQ-TREE v1.6.12 with the best-fit model WAG+I+G4. A maximum-likelihood phylogenomic tree was also constructed using FastTree v2.1.10 [39] and visualized with ITOL after identifying and aligning a concatenated set of 120 marker proteins with GTDB-Tk v1.7.0 [40]. The genomic average nucleotide identity (gANI) was calculated using the average nucleotide identity based on BLAST (ANIb) algorithm implemented in pyani v0.3.0-alpha (Python 3.8.15) [41] using BLAST+ 2.13.0 with the default fragment length of 1020 bp. Only reciprocal alignments that covered ≥70% of the genome and showed ≥30% nucleotide identity were retained for the final matrix.

### Statistical analysis

The influence of soil depth on soil properties was assessed using one-way analysis of variance (ANOVA). Differences among means were compared using Duncan's multiple range test in SPSS (Statistical Package for the Social Sciences, version 20.0; SPSS Inc., Chicago, IL, USA), with a *P*-value <.05 considered statistically significant. Spearman correlation analyses among the relative abundance of USCγ, *pmoA* gene abundance, and soil physicochemical properties were also conducted in SPSS [42].

## Results and discussion

### Methanotroph abundance and composition in situ

**Figure 1:**
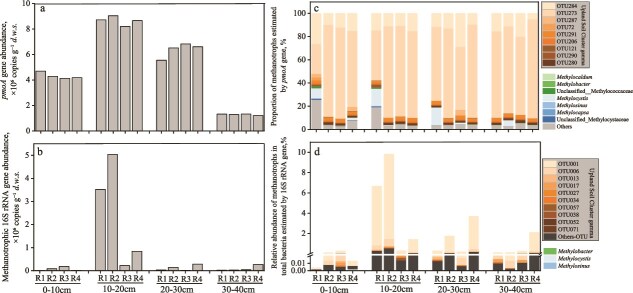
Methanotrophic community compositions and abundance in soil in situ. (a) Methanotrophic abundance based on *pmoA* gene copy numbers. (b) Abundance of methanotrophs among total microorganisms according to 16S rRNA gene copy numbers. (c) Methanotrophic community composition based on amplicon sequencing of the *pmoA* gene. (d) Relative abundance of methanotrophs among total microorganisms and their community composition based on amplicon sequencing of the 16S rRNA gene.

The abundance of methanotrophs varied across soil depths and peaked significantly in the 10–20 cm layer (Fig. 1A, B). Based on the *pmoA* gene, abundances ranged from 1.21 × 10^6^ to 9.04 × 10^6^ copies g^−1^ dry weight soil (*d.w.s.*) (Fig. 1A), consistent with previously reported values for grassland soils [43, 44]. The corresponding 16S rRNA gene abundance (estimated by MiSeq sequencing and qPCR) ranged from 0.35 × 10^4^ to 5.04 × 10^6^ copies g^−1^  *d.w.s.* (Fig. 1B). Amplicon sequencing of the *pmoA* gene revealed that USCγ was the dominant methanotroph from the surface to 40 cm depth, accounting for 75.71%–99.62% of the total methanotrophs. A total of 26 USCγ OTUs were detected, among which OTU273 was the dominant phylotype (25.60%–85.25%; Fig. 1C). Previous methanotroph surveys in karst caves have demonstrated the substantial diversity of the USCγ clade [45]. Similarly, 16S rRNA gene amplicon sequencing identified 479 USCγ OTUs, which accounted for 6.68% and 9.83% of the total microorganisms in the 10–20 cm layer at sites R1 and R2, respectively (Fig. 1D). Therefore, metagenomic sequencing was performed on these two samples.

The physicochemical properties of the soil layers examined in this study, including particle size composition, pH, soil moisture, and inorganic nitrogen concentration (Table 1), showed no significant correlation with the enrichment of USCγ in the 10–20 cm layers (Table S1). Thus, other factors, such as oxygen concentration, soil porosity, and methane diffusion rate, likely play key roles in shaping the vertical distribution pattern of USCγ [7, 46, 47]. In this study, soil moisture increased with depth, and the silt fraction accounted for ~45% of the particle size composition, giving the soil an intermediate gas diffusion capacity between sandy and clay soils [48]. Both porosity and the diffusive fluxes of methane and oxygen decreased with depth, shifting the micro-oxic–anoxic interface upward; this upward shift likely explains the peak in USCγ abundance observed at 10–20 cm [46].

### Reconstruction of USCγ MAGs

We obtained six USCγ MAGs, with completeness ranging from 79.89% to 91.70% and contamination levels ranging from 0.06% to 1.81%. GTDB-Tk classified all MAGs into the genus JACCXJ01 (Gammaproteobacteria), which is currently a provisional placeholder in the GTDB database and lacks formal nomenclature [49] (Table 2). Among these, MAG Bin.182 contained the intact 16S, 5S, and 23S rRNA genes as well as the complete *pmoCAB* operon. It was therefore selected for further analysis and designated USC_AKS (where “AKS” abbreviates the sampling county). A comparison of USC_AKS with other published USCγ MAGs revealed that its genome size (3.51 Mb) and number of coding genes (3601) are within the medium range. Regarding GC content, the eight MAGs could be visually grouped into three distinct ranges based on natural breakpoints. USC_AKS and MGR_bin175 exhibited the highest GC content (64.38% and 63.94%, respectively), followed by five MAGs with values in the range of 59.50%–60.50%. H1-B1_maxbin2.bin.5_sub showed the lowest GC content, at 57.88% (Table S2).

**Table 2:** Genome statistics of the six MAGs obtained in this study. USC_AKS was derived exclusively from Bin.182.

Soil sample	166-R1(10–20 cm)			170-R2(10–20 cm)			
Raw data size, G	30			30			
Number of clean reads	508 144 798			481 311 862			
MAG	Bin_298	**Bin_182**		Bin_012	Bin_090	Bin_014	Bin_247
Assembly method	metaSPAdes	MEGAHIT		metaSPAdes	metaSPAdes	MEGAHIT	MEGAHIT
Binning method	MetaBAT2	MetaBAT2		MaxBin2	MetaBAT2	MaxBin2	MetaBAT2
Size, M	3.91	3.51		2.59	2.36	3.06	2.56
Read count	579 537	890 643		801 222	613 548	1 174 018	714 964
The percentage of reads mapped to bin, %	0.11	0.18		0.17	0.13	0.14	0.15
Coding density	1.02	1.03		1.03	0.99	1.09	1.07
Completeness, %	91.7	89.08		83	80.6	81.84	79.89
Contamination, %	1.47	1.76		0.55	0.06	1.66	1.81
Taxa	o__JACCXJ01	o__JACCXJ01		o__JACCXJ01	o__JACCXJ01	o__JACCXJ01	o__JACCXJ01
GC content, %	64.96	64.38		65.64	65.79	64.70	65.40
Number of contigs	384	299		427	327	471	326
Number of genes	3081	3601		2664	2360	3357	2735
Number of tRNAs	39	45		34	32	32	32
rRNA operon	16S(1)	5S(1),16S(1),23S(1)		16S(1)	16S(1)	-	-

### Phylogeny of USC_AKS

The *pmoA* gene of USC_AKS is 744 bp in length, identical in size to those in the reported MAGs (USCγ_Taylor [15], MGR_bin175 [16], Lehman Cave wb1-P19 and Frasassi Cave wb1-P19 [17], H1-B1_maxbin2.bin.5_sub, SD8020_metabat2.bin.3, and SD8037_metabat2.bin.6 [18]). This length also matches that of most Type I methanotroph strains, such as *Methylobacter tundripaludum* SV96 [50] and *Methylocaldum sp.* SAD2 [51] (Fig. S1). In contrast, most Type II methanotrophs have a *pmoA* gene length of 759 bp.

**Figure 2:**
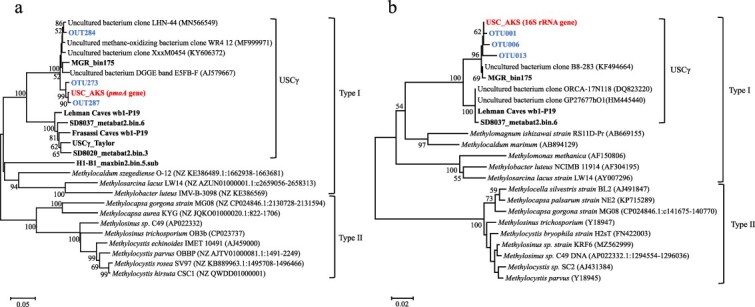
Phylogenetic relationship of USC_AKS with its relatives based on full-length and representative sequences obtained from the *pmoA* (a) and 16S rRNA (b) genes. Neighbor-joining trees were constructed using MEGA 7.0 with 1000 bootstrap replicates. Only bootstrap values above 50% are shown at the branch nodes. Scale bars indicate 0.05 or 0.02 substitutions per nucleotide position. The red-labeled USC_AKS represents the full-length *pmoA* and 16S rRNA gene sequences derived from the MAG reconstructed in this study. The blue-labeled OTUs correspond to *pmoA* and 16S rRNA gene sequences obtained via amplicon sequencing in this study. Sequences shown in bold black are full-length *pmoA* and 16S rRNA gene sequences retrieved from eight USCγ MAGs.

The full-length *pmoA* gene sequence of USC_AKS exhibits the highest homology with that of MGR_bin175, with which it forms an independent USCγ subclade. It shows 96.18% identity to the representative upland soil sequence (AJ579667) originally used to define the USCγ clade (Fig. 2A). The full-length 16S rRNA genes from USC_AKS, MGR_bin175, Lehman Cave wb1-P19, and SD8037_metabat2.bin.6, together with all representative USCγ OTUs, cluster into a single, well-supported clade that contains no cultivated strains (Fig. 2B).

BLAST analysis of the full-length 16S rRNA gene of USC_AKS against the NCBI database revealed that the highest nucleotide identities (>97%) were to uncultured bacteria from environmental samples (accession numbers: JN672117, HM445440, and KC569827) (Fig. 2B). In contrast, the identity to *Thioalkalivibrio sulfidiphilus* HL-EbGr7 (CP001339) [52], a member of the order Chromatiales, was only 93.03% (with 100% query coverage), which was not the highest among all BLAST hits. This result is consistent with an earlier online BLASTn search of the 488-nt 16S rRNA fragment from USCγ_Taylor, which showed 94% identity to Chromatiales members *Thioalkalivibrio* (NR_074692) and *Ectothiorhodospira* (NR_125567) [15]. Instead, the latest GTDB taxonomy classifies USC_AKS within the genus JACCXJ01 (Table 2). Similarly, the four methanotrophs reported by Bay *et al.* [18] all fall within the USCγ/JACCXJ01 clade, with no mention of a Chromatiales-related lineage. In the study by Jones *et al.* [17], genome-based taxonomy assigns wb1-P19 to USCγ, and under the newly proposed nomenclature, USCγ is placed in the order “*Candidatus* Methyloligotrophales”. To date, the precise taxonomic placement of USC remains uncertain and will require clarification once a pure strain is obtained.

**Figure 3:**
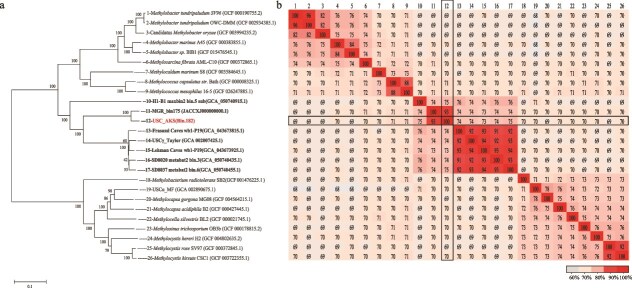
Phylogenetic relationships and pairwise genome sequence similarity between USC_AKS and its relatives. (a) Maximum-likelihood phylogenetic tree showing the placement of USC_AKS (Bin182, in red) within the USCγ clade of representative reference genomes. The remaining MAGs are shown in bold black. Bootstrap values were calculated from 1000 replicates. Scale bar equals 0.10 amino-acid substitutions per site. (b) Matrix of pairwise gANI values among all strains, presented in the same order as in (a). The intensity of red color scales with the degree of nucleic acid sequence similarity between genomes.

A phylogenomic tree shows that the eight available USCγ MAGs form a well-supported monophyletic clade, which clusters within Type I methanotrophs, confirming that USCγ belongs to this group (Fig. 3A). Within this main USCγ clade, three subclades can be distinguished, consistent with the groupings based on GC content (Table S2). The gANI among MAGs within each subclade was consistently below 95% (Fig. 3B). Based on the GTDB taxonomic criterion for species delineation (gANI >95%), these eight USCγ MAGs therefore represent eight distinct species. This finding highlights the substantial phylogenetic diversity contained within this as-yet-uncultivated methanotrophic lineage.

### Methane oxidation pathway of USC_AKS

**Figure 4:**
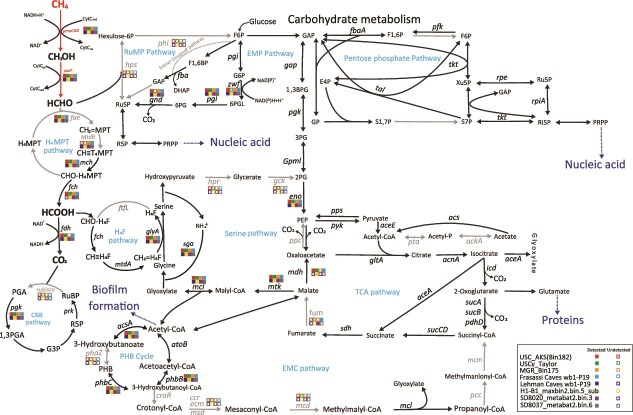
Metabolic reconstruction of USC_AKS. Pathways are drawn based on KEGG map files and KO assignments. Black and gray dashed arrows indicate the presence and absence, respectively, of related genes in the USC_AKS MAG. Dotted arrows represent multiple enzymatic reactions. Abbreviations: RuMP, ribulose monophosphate; EMP, Embden–Meyerhof–Parnas; GAP, glyceraldehyde-3-phosphate; PHB, poly-β-hydroxybutyrate; H_4_F, tetrahydrofolate; H_4_MPT, tetrahydromethanopterin; EMC, ethylmalonyl-CoA; TCA, tricarboxylic acid; CBB, Calvin–Benson–Bassham. Additionally, key genes in the USC-related MAGs are annotated using a heatmap.

Aerobic methanotrophs catalyze methane oxidation in soil through a sequential enzymatic cascade: methane is first oxidized to methanol by methane monooxygenase (MMO), then successively converted by methanol dehydrogenase, formaldehyde dehydrogenase, and formate dehydrogenase into formaldehyde, formate, and ultimately CO_2_, with energy generated via electron transfer. Based on KEGG functional annotation of eight MAGs, we reconstructed the core metabolic pathways of USC_AKS and other USCγ-related methanotrophs (Fig. 4). All MAGs analyzed in this study contained a complete *pmoCAB* operon encoding particulate methane monooxygenase (pMMO), but none possessed genes for soluble methane monooxygenase (sMMO) or related variants such as *pxmA* and *pmoA*2 (Fig. 4, Table S3). This result is consistent with the characteristics of all previously reported USCα MAGs to date, including *Candidatus* Methyloaffinis lahnbergensis [3], *Methylocapsa gorgona* MG08 [53], *Methylocapsa* sp. Bog 942/Bog 920 [54], and USCα-AHI [55]. Although no *mxaF*-type methanol dehydrogenase was detected in USC_AKS, a highly active homodimeric methanol dehydrogenase encoded by *xoxF* was identified and was also commonly found in the other seven MAGs (Table S3) [56]. Phylogenetic analysis further indicated that the *xoxF* gene detected in USC_AKS and the other MAGs belongs to the *xoxF5* clade (Fig. S2). As a PQQ-dependent, rare-earth-element-requiring enzyme, *xoxF* must transfer electrons to cytochrome c, and the reoxidation of cytochrome c strictly depends on O_2_ as the terminal electron acceptor, rendering the process obligately aerobic [56]. Genes encoding enzymes responsible for the sequential oxidation of formaldehyde to formate and ultimately to CO_2_ were largely complete across all eight MAGs. The absence of specific genes in a few MAGs is most likely attributable to gaps in genome assembly rather than genuine metabolic deficiencies. Although USC-related MAGs harbor near-complete gene sets for CH_4_-to-CO_2_ oxidation and phylogenetically cluster with uncultured atmospheric methanotrophs—traits consistent with a high-affinity methanotrophic lifestyle—functional validation via in situ activity assays and kinetic measurements remains essential.

### Carbon assimilation by USC_AKS

Phylogenetic analysis classifies USC_AKS as a Type I methanotroph, which typically assimilates formaldehyde—a central intermediate in methane oxidation—through the ribulose monophosphate (RuMP) pathway [57]. However, USC_AKS lacks the genes encoding two key enzymes of the RuMP pathway—3-hexulose-6-phosphate synthase (*hps*) and 6-phospho-3-hexuloisomerase (*phi*) (Fig. 4, Table S3). When the first USC genome (USCγ_Taylor) was reported in 2017, the authors already noted the absence of these key enzymatic genes [15]. Notably, we found that the absence of these two key genes is conserved in subsequently reported USCγ MAGs from diverse geographical locations, appearing to be a common feature of this lineage. This distinguishes USCγ from canonical Type I methanotrophs [58]. Whether the functions of these two enzymes have been replaced by unknown enzymes or whether their coding genes were simply missed during genome assembly in the USCγ lineage remains inconclusive. Furthermore, genes encoding enzymes for multiple key steps in the Embden–Meyerhof–Parnas (EMP) pathway and the pentose phosphate pathway (PPP) were detected in these USCγ MAGs (Fig. 4, Table S3).

In the initial report of the first USCγ genome (USCγ_Taylor), the authors originally described a complete serine cycle but later issued a correction, revising the statement to the more ambiguous claim that it “contains nearly all genes required for the serine biosynthesis pathway” [15]. The serine pathway is utilized by Type II methanotrophs for carbon assimilation via formate. The serine cycle in USC_AKS is also incomplete, lacking the hydroxypyruvate reductase gene (*hpr*) and all genes encoding enzymes required for the conversion of glycerate to 2-phosphoglycerate (2PG) (Fig. 4, Table S3). This finding contradicts earlier reports describing that the wb1-P19 MAGs from Lehman and Frasassi caves possess a complete set of serine pathway genes for formaldehyde metabolism [17]. Our re-analysis indicates that all eight MAGs analyzed lack *hpr* and consequently have an incomplete serine cycle (Table S3). This discrepancy most likely reflects successive updates to the KEGG database and/or differences in annotation pipelines; however, genuine biological novelty cannot be ruled out. Future studies should adopt complementary approaches, including isolation cultivation and targeted transcriptomics, to explore and validate the relevant metabolic pathways.

Glyoxylate regeneration is a key step for carbon assimilation in the serine cycle [59]. In USC_AKS, the *aceA* gene, encoding isocitrate lyase—the hallmark enzyme of the glyoxylate shunt—is present, and the tricarboxylic acid (TCA) cycle gene set is largely complete (Fig. 4, Table S3). This implies that acetyl-CoA produced by the serine cycle (if this pathway is complete in USCγ) could be oxidized to glyoxylate via the glyoxylate shunt, thereby ensuring the continuous operation of the serine cycle. Such a “glyoxylate regeneration route” is characteristic of Type IIb methanotrophs but is rarely encountered in Type IIa methanotroph lineages. However, USC_AKS lacks the four core enzymes of the ethylmalonyl-CoA (EMC) pathway, indicating that glyoxylate cannot be regenerated via this route as in Type IIa methanotrophs [60]. We also examined the eight USCγ MAGs for the reductive glycine pathway (rGlyP) proposed by Tveit *et al.* [53] for *Methylocapsa gorgona* MG08. However, none of the MAGs contained a complete set of the core rGlyP genes (e.g. *gcvP, gcvT, gcvH, lpd, folD, glyA*), suggesting that this pathway is unlikely to contribute to carbon assimilation in USCγ. Therefore, we suspect that USC_AKS employs an as-yet-unidentified pathway for carbon assimilation, although conclusive evidence is still lacking.

Acetyl-CoA produced in the serine cycle can also be converted to poly-beta-hydroxybutyrate (PHB) (Fig. 4), a carbon-storing polymer that serves as an endogenous source of reducing power for methanotrophs [61]. This capability may help USC_AKS adapt to environments with fluctuating substrate supplies [62, 63]. In addition, USC_AKS lacks Rubisco, the key CO_2_-fixing enzyme of the CBB cycle, a deficiency that aligns with observations for USCγ_Taylor, the wb1-P19 MAGs from Lehman and Frasassi caves, and MGR_bin175 [15–17]. Our re-analysis further confirms the absence of Rubisco in all examined USCγ MAGs (Table S3), thereby ruling out autotrophic carbon fixation via the CBB pathway in this lineage.

### Biofilm formation by USC_AKS

Enriching the USCγ clade remains a significant challenge, yet obtaining pure cultures or stable enrichments is the most direct way to elucidate their physiology and metabolism. Notably, USCγ and USCα have traditionally been considered incapable of growth under elevated methane concentrations. A draft genome of a forest soil USCα MAG revealed a high abundance of genes involved in extracellular polysaccharide biosynthesis, a functional trait associated with biofilm formation. This genomic evidence suggests that USCα may depend on a biofilm growth mode, which could explain its resistance to conventional enrichment and cultivation methods [13]. Subsequently, Tveit *et al.* [53] successfully isolated the first USCα strain, *Methylocapsa gorgona* MG08, by filtering soil cells onto polycarbonate membranes, floating them on 10-fold diluted Nitrate Mineral Salts medium, and incubating them under 20% CH_4_.

Given the ecological parallels between USCα and USCγ, we screened the USC_AKS genome for biofilm-associated genes and identified a near-complete regulatory suite that includes intracellular signaling components (e.g. *crp, cpdA, chpA, pilG*) and extracellular polysaccharide (EPS) biosynthesis genes (e.g. *weaE, gtE, wbpW, wecB*). These genetic elements are predicted to coordinately function in assembling a multicellular biofilm matrix external to the cell (Table S4). Based on these genomic insights, the membrane-based enrichment strategy that succeeded for USCα may also prove promising for isolating USCγ in pure culture from grassland soils and cave environments, although its effectiveness remains to be empirically tested.

### Nitrogen and sulfur metabolism by USC_AKS

No genes encoding the structural core of nitrogenases (*nifHDK, vnfHDK, anfHDK*) were found in USC_AKS or the other examined USCγ MAGs, while accessory genes like *nifA, nifU*, and *nifQ* were present (Fig. S3, Table S3). The lack of essential catalytic components indicates that biological N_2_ fixation is non-functional in USCγ. Consequently, its nitrogen metabolism must depend on exogenous combined nitrogen sources (e.g. NH_4_^+^, NO_3_^−^). The retained accessory genes may be evolutionary relics or may be constitutively expressed for other cellular roles, such as Fe-S cluster assembly. Unlike the widespread N_2_-fixation capacity characteristic of Type II methanotrophs (e.g. *Methylosinus trichosporium* OB3b and *Methylocystis* sp. strain SC2) [64, 65], genomic or physiological evidence for N_2_ fixation has thus far been reported only in a limited subset of Type I methanotrophs, such as certain species of *Methylococcus* and *Methylomonas*, as well as the strain *Methylobacter marinus* A45 [64, 66]. The genomic information from this study also revealed the presence of genes for assimilatory nitrate reduction (*nasA* and *nirA*), heterotrimeric nitrite reduction to ammonium (*nirBD*), ammonium transporter protein (*amt*), and nitrate/nitrite transporter protein (*nrt*) (Fig. S3, Table S3). The presence of these genes suggests that USC_AKS has the potential to utilize a wide range of combined nitrogen sources. Genes encoding the denitrification pathway were absent in USC_AKS, a feature shared with some other aerobic methanotrophs [67].

Both USC_AKS and other USCγ MAGs lack canonical energy-conserving sulfur-oxidizing modules (e.g. *SoxCD, AprA, SoeABC*) and complete sulfate-respiratory machinery (e.g. *DsrAB, QmoABC*) (Table S3). However, they carry a set of genes involved in sulfur assimilation (Table S3), including *cysH* and *cysQ*, which catalyze later steps of sulfate reduction to sulfide, as well as genes related to methionine biosynthesis (*metA, metZ*) and to the synthesis of sulfur-containing amino acids from sulfide (*cysE*). Notably, USC_AKS also contains genes involved in sulfur metabolism, including the sulfite reductase gene cluster (*sir*), thiosulfate/3-mercaptopyruvate sulfurtransferase (*sseA*), and sulfate adenylyltransferase (*sat*). However, these genes are not specific to sulfur oxidation pathways and may be bidirectional or serve other cellular functions. An important distinction is that the seven previously reported MAGs carry *cysUWA*, encoding components of the sulfate/thiosulfate transport system (permease and ATP-binding proteins), whereas this operon is absent in USC_AKS. To date, research on sulfur metabolism in aerobic methanotrophs remains limited [68]. The potential interplay between sulfur metabolism and carbon metabolism, energy acquisition, and environmental adaptation in methanotrophs warrants further investigation using transcriptomic and other omics-based approaches in future studies.

### Ecological and physiological properties of USC_AKS

A BLASTn search of the full-length 16S rRNA gene from USC_AKS against GenBank identified 57 sequences with >95% identity, predominantly from lava caves (55 sequences, 96.50%), with single sequences from tall-grass prairie soil and a permafrost core. These results highlight lava caves as a major habitat for USCγ, consistent with reports of their high relative abundance in cave ecosystems globally [69, 70]. For instance, USCγ accounted for 17–22% of microbial communities in karst caves across Guilin, China [69], and ~20% in Heshang Cave, Hubei Province [71]. Similarly, USCα comprised up to 10% of the total 16S rRNA gene sequences in a Portuguese cave dataset [13]. Together, these findings demonstrate that atmospheric methanotrophs such as USCγ and USCα do not require vegetated or soil-rich ecosystems, but rather thrive in mineral-dominated subterranean environments [72]. Currently, USCγ MAGs from Lehman Cave wb1-P19, Frasassi Cave wb1-P19, H1-B1_maxbin2.bin5_sub, SD8020_metabat2.bin.3, and SD8037_metabat2.bin.6 have been obtained from limestone caves in Great Basin National Park, Nevada, USA [17], Italy, and Australia [18], respectively. Their widespread occurrence in caves further suggests that USCγ-type methanotrophs may function as pioneer colonists on newly formed rock surfaces [73, 74], independent of plant-derived carbon or developed soils.

Moreover, temperature and moisture are unlikely to be key determinants of USCγ distribution. For example, the MAGs USCγ_Taylor and MGR_bin175 were retrieved from permafrost in Taylor Valley and the Mackay Glacier in Antarctica [15, 16], while USC_AKS was obtained from a temperate desert grassland. In some karst caves of China, USCγ is frequently enriched not only on cave walls but also in underlying sediments [71]. Together, these habitats span an extensive thermal gradient and highly contrasting moisture conditions, demonstrating that temperature and water availability are not decisive factors controlling the distribution and persistence of USCγ. At present, pH appears to be the sole environmental factor that clearly governs the geographic distribution of USCγ. The habitats of MGR_bin175 (pH = 7.47–10.24), USCγ_Taylor (pH = 8.15), H1-B1_maxbin2.bin.5_sub, SD8020_metabat2.bin.3 and SD8037_metabat2.bin.6 (pH = 8.05–8.34), and USC_AKS (pH = 8.76, Table 1) are overwhelmingly alkaline, consistent with earlier findings [3]. However, several studies have reported significant depth-related shifts in the relative abundance of USCγ along soil profiles [75], implying that—beyond pH—potential factors such as O₂ concentration, particle size distribution, soil porosity, and carbon and nitrogen contents, or trace metal composition may also exert control; these possibilities remain to be rigorously tested.

All published USCγ MAGs lack the signature enzymes of the RuMP pathway—the dominant carbon assimilation pathway in their phylogenetic group, Type I methanotrophs—as well as Rubisco from the CBB pathway [56] (Table S2). This absence suggests two possible explanations: on the one hand, USCγ may oxidize methane without assimilating methane-derived carbon, relying instead on alternative carbon sources such as acetate. On the other hand, the observed truncation of known carbon assimilation pathways in these MAGs could result from incomplete genome assembly rather than genuine metabolic deficiencies. Additionally, we speculate that USCγ may employ an alternative methane assimilation route distinct from the canonical RuMP and serine pathways. Although Edwards *et al.* [15] reported the first assembled genome of USCγ, subsequent studies have not explicitly proposed that USCγ might possess unidentified carbon fixation pathways. Therefore, obtaining pure cultures remains the most effective and essential approach to clarifying the metabolic pathways of USCγ and should be a central focus of future research.

### Advances in understanding of USCγ clade

This study advances the field in two key areas: phylogenetic analysis and metabolic pathway reconstruction. First, we report the first USCγ MAG (USC_AKS) derived from a typical temperate grassland ecosystem, thereby expanding current knowledge of the geographical distribution and phylogenetic diversity of this clade. A phylogenetic tree constructed from whole-genome sequences revealed that the existing USCγ MAGs can be divided into three independent subclades with distinct genetic differences. Within this framework, USC_AKS represents a grassland-specific clade that is clearly distinguishable from MAGs originating from other specialized habitats, such as polar permafrost and caves.

Second, through systematic comparative genomic analysis of eight USCγ MAGs from different geographic origins, this study provides, for the first time at the genomic level, conclusive evidence of a consistent and conserved deficiency in the carbon assimilation pathways of this clade. Our results demonstrate that none of the known USCγ MAGs possess a complete conventional carbon assimilation pathway. Specifically: (i) they lack the core enzyme genes (*hps* and *phi*) of the RuMP pathway, which is typical of Type I methanotrophs; (ii) they lack a complete serine pathway, including the hydroxypyruvate reductase gene (*hpr*) and genes encoding enzymes required for the conversion of glycerate to 2-phosphoglycerate—a pathway typically used by Type II methanotrophs; and (iii) they lack the gene encoding Rubisco, the key carbon-fixing enzyme of the CBB cycle. These three metabolic deficiencies are consistently present across all eight USCγ MAGs analyzed, confirming the universality of this finding.

In summary, this study elevates previous preliminary observations or speculations based on individual MAGs to a definitive conclusion validated across multiple MAGs. Our findings clearly demonstrate that the USCγ clade lacks all known conventional carbon assimilation pathways, suggesting that USCγ may employ a previously unidentified carbon fixation strategy. This discovery provides a critical genomic foundation for understanding the energy metabolism and survival mechanisms of atmospheric methane-oxidizing bacteria in oligotrophic environments and establishes clear directions for future research aimed at elucidating their unique carbon assimilation pathways through pure culture isolation, transcriptomics, and metabolic flux analysis.

## Conclusions

Based on metagenomic sequencing of a USCγ-rich desert grassland soil, we obtained the first grassland-derived USCγ MAG. An integrated analysis with other USCγ MAGs from polar and cave environments revealed that USCγ exhibits substantial phylogenetic diversity, comprising at least three subclades and eight genomes at the species level. While these MAGs encode a complete pathway for methane oxidation to CO₂, they lack the complete carbon assimilation pathways typical of cultured methanotrophs, including the RuMP pathway, the CBB cycle, and the serine cycle. Given the absence of key genes from the conventional RuMP and serine pathways, we speculate that USCγ may employ an alternative methane assimilation route. These results suggest that USCγ utilizes a carbon metabolism distinct from known methanotrophs, potentially involving an as-yet-uncharacterized carbon-assimilation mechanism that warrants further investigation. Metabolic predictions based on these MAGs further indicate that USCγ employs a rare-earth-element-dependent *XoxF*-type methanol dehydrogenase, lacks N_2_-fixing capability, and harbors genes involved in biofilm formation and PHB metabolism. These metabolic features not only offer an intrinsic explanation for their resistance to cultivation but also provide key theoretical guidance for designing targeted enrichment and isolation strategies in future studies.

## Supplementary Material

Supplementary_material_ycag151

## Data Availability

The raw amplicon sequence datasets for the 16S rRNA and *pmoA* genes have been deposited at the NCBI Sequence Read Archive (SRA) under BioProject number PRJNA1265376 and PRJNA1265377. This whole-genome shotgun project and the USC_AKS draft genome have been deposited in DDBJ/ENA/GenBank under accession number SRP589396 (Sequence data will be released upon article publication). The full-length 16S rRNA and *pmoA* gene sequences extracted from the MAGs were submitted to GenBank: BankIt under accession number Submission ID 2967339 and 2 967 355. All the data generated or analyzed during this study are included in this published article and its supplementary information files.
