# Supplementary material for: Molecular insights into atmospheric methane-oxidizing USCγ from desert grassland soil based on metagenome-assembled genome analysis
Source: ISME Commun. 2026 Jun 3;6(1):ycag151. doi: 10.1093/ismeco/ycag151 (PMC13333085; doi:10.1093/ismeco/ycag151)
Supplement: Supplementary_material_ycag151 [file supplementary_material_ycag151.zip › Supplementary_Tables_and_Figures_ycag151.docx]

**Supplementary Information for**

**Molecular Insights into Atmospheric Methane‐oxidizing USCγ from Desert Grassland Soil Based on Metagenome-assembled Genome Analysis**

Yufang Wang^1,2,3^, Yuanfeng Cai^1*^, Zechen Peng ^3^, Fujiang Hou^3^, Zhongjun Jia^1,4^

**Correspondence author:** Dr. Yuanfeng Cai, State Key Laboratory of Soil and Sustainable Agriculture, Institute of Soil Science, Chinese Academy of Sciences, No. 298, Chuangyou Road, Qilin Street, Jiangning District, Nanjing, Jiangsu Province, 211135, P.R. China.

E-mail: yfcai@issas.ac.cn

**This file includes:**

Figures S1 to S3

Tables S1 and S2

**Other supplementary materials for this manuscript include the following:**

Tables S3 and S4, please see separate Excel files.

**Table S3.** KEGG annotation results of genes in USC_AKS and the previously reported USCγ MAGs.

**Table S4.** KEGG annotation results of genes and metabolic pathways involved in biofilm formation in USC_AKS and previously reported USCγ MAGs.


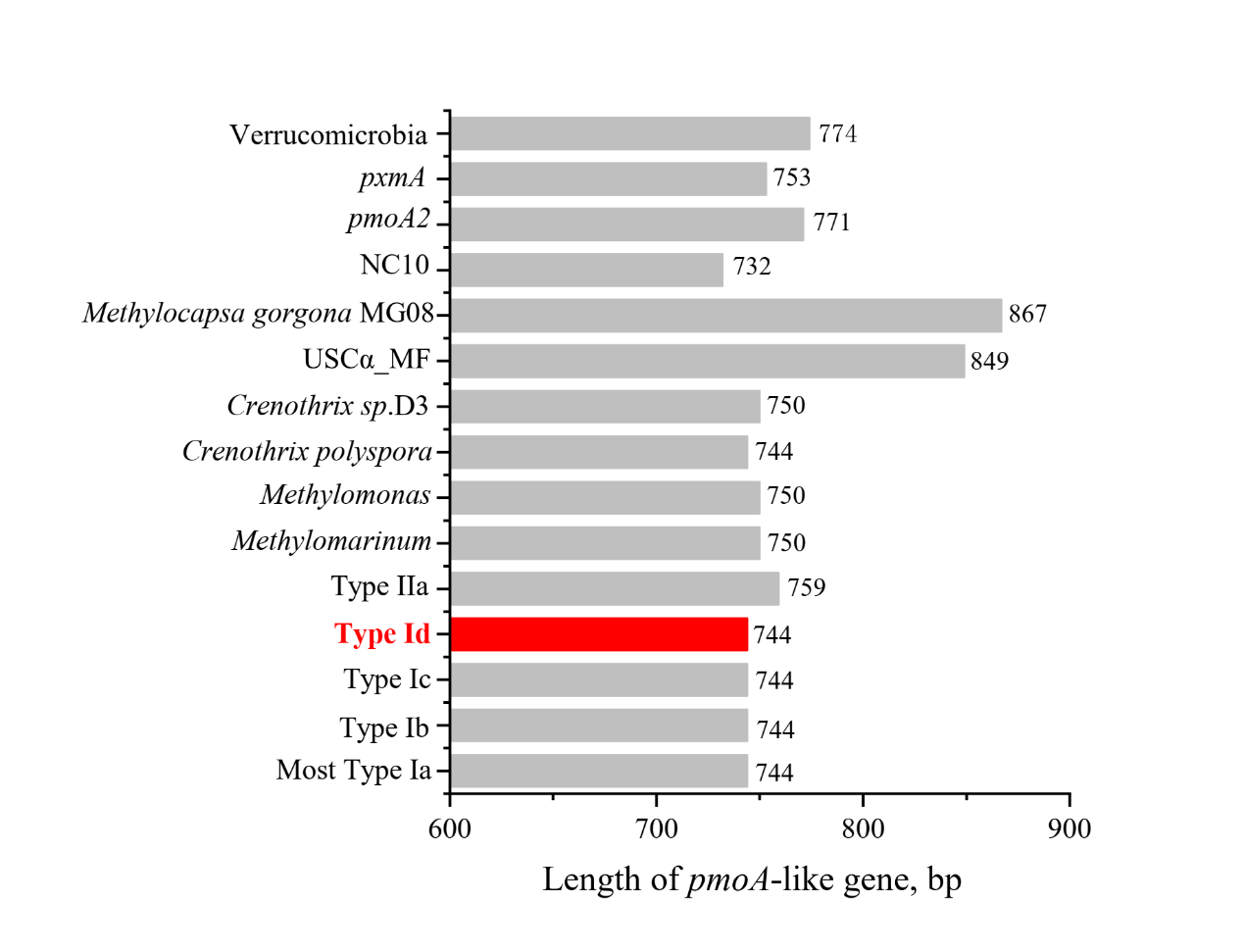
**Fig.** **S1.** Length of *pmoA*-like genes in known methanotrophs. USC_AKS is classified as a Type I methanotroph. Lengths of Verrucomicrobia *pmoA* genes are shown as average values for multiple *pmoA* copies from several strains. According to the current genome sequence version (NZ_CP019948.1, 12-APR-2021), the *pmoA*2 gene of *Methylocystis bryophila* S285 is 762 bp in length. Type Id includes USC_AKS and currently reported USCγ members: Frasassi Caves wb1-P19, H1-B1_maxbin2.bin.5_sub, Lehman Caves wb1-P19, SD8037_metabat2.bin.6, SD8020_metabat2.bin.3, MGR_bin175 and USCγ_Taylor. Further details can be found in Table S5 of Cai et al., 2022 [1].


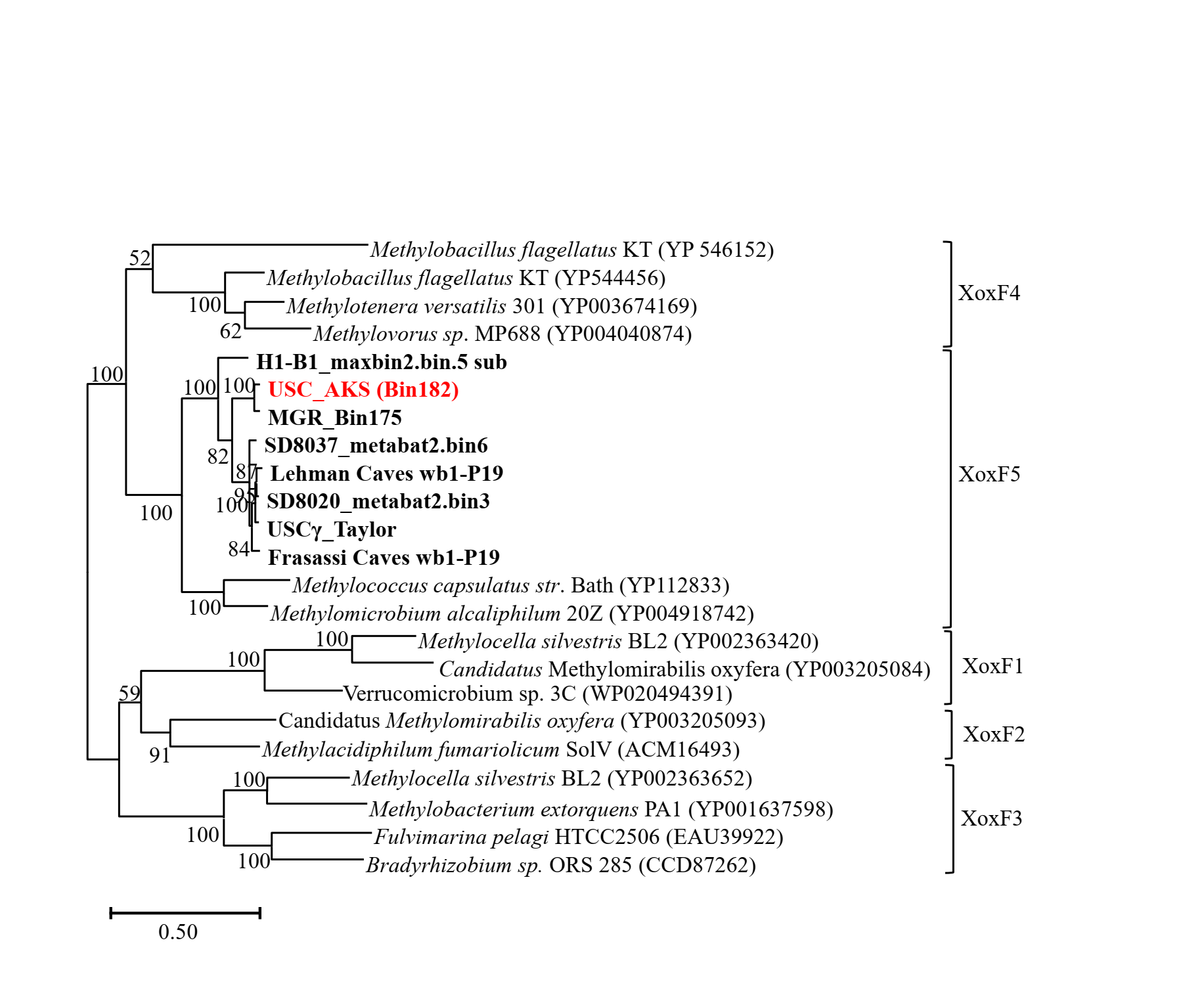


**Fig. S2.** Phylogenetic tree of the XoxF protein sequence deduced from the *xoxF* gene sequences of USC_AKS and its relatives. The scale bar represents 0.50 substitutions per amino acid position. The red-labeled USC_AKS indicates the XoxF sequences derived from the MAG reconstructed in this study. Sequences shown in bold black represent XoxF sequences retrieved from published USCγ MAGs.


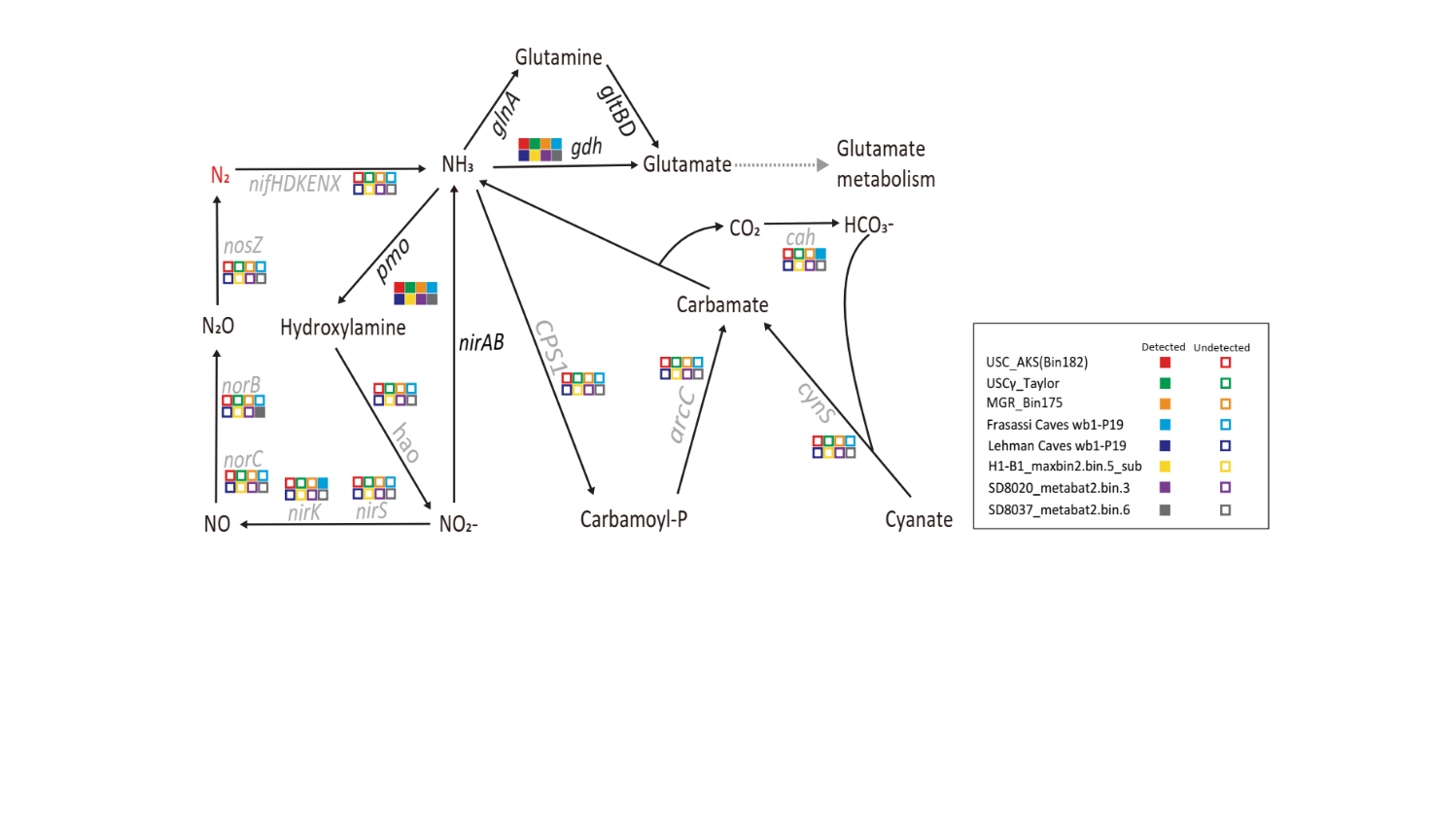
**Fig. S3.** Nitrogen metabolic genes in USC_AKS and the other seven reported USCγ MAGs, as annotated via the KEGG database.

Table S1 Spearman correlations of USCγ relative abundance and *pmoA* abundance versus soil physicochemical properties.

|  | USCγ relative abundance, % | *pmoA* abundance, copies g⁻¹ *d.w.s.* |
| --- | --- | --- |
| Particle size composition | |  |
| <5 μm | -0.286 | -0.118 |
| 5–10 μm | 0.23 | -0.112 |
| 10–50 μm | 0.333 | 0.038 |
| 50–100 μm | -0.137 | 0.05 |
| 100–500 μm | -0.035 | 0.172 |
| 500–1500 μm | 0.147 | 0.184 |
| pH | 0.218 | -0.065 |
| Soil Moisture, % | 0.456 | -0.076 |
| NO_3_^-^–N, mg kg^-1^ | -0.059 | -0.132 |
| NH_4_^+^–N, mg kg^-1^ | -0.294 | -0.006 |
| Total Nitrogen, g kg^-1^ | -0.138 | -0.179 |
| Total Carbon, g kg^-1^ | -0.159 | -0.044 |

Note: *Significant at the 0.05 level (two-tailed). **Significant at the 0.01 level (two-tailed).

| Sample Name | **USC_AKS**  **(Bin_182)** | MGR_Bin175 | USCγ_Taylor | Lehman Caves  wb1-P19 | Frasassi Caves wb1-P19 | H1-B1_max  bin2.bin.5_sub | SD8020_metabat2.bin3 | SD8037_metabat2.bin6 |
| --- | --- | --- | --- | --- | --- | --- | --- | --- |
| Size, Mb | 3.51 | 4.57 | 2.96 | 2.38 | 4.56 | 5.8 | 4.15 | 3.63 |
| Completeness, % | 89.08 | 93.11 | 89.99 | 89.98 | 91.55 | 92.05 | 92 | 93.43 |
| Contamination, % | 1.76 | 5.3 | 1.11 | 0.34 | 4.12 | 3.36 | 3.97 | 0.23 |
| Taxa | o__JACCXJ01 | o__JACCXJ01 | o__JACCXJ01 | o__JACCXJ01 | o__JACCXJ01 | o__JACCXJ01 | o__JACCXJ01 | o__JACCXJ01 |
| GC Content, % | 64.38 | 63.94 | 60.03 | 60.41 | 59.65 | 57.88 | 59.73 | 59.94 |
| Number of Contigs | 299 | 417 | 246 | 233 | 415 | 1403 | 391 | 130 |
| Number of genes | 3601 | 4981 | 3069 | 2369 | 4770 | 7812 | 4421 | 3711 |
| Number of tRNA | 45 | 46 | 36 | 40 | 43 | 48 | 38 | 46 |
| rRNA operon | 5S(1),16S(1),23S(1) | 16S(1),5S(1) | 16S(1),5S(1),23S(1) | 16S(1),5S(1),23S(1) | - | 23S(1) | - | 16S(1),5S(1),23S(1) |

Table S2. Genome statistics of the MAG USC_AKS and the seven reported MAGs of USCγ.

**Reference**

[1] Cai YF, Yun JL, Jia ZJ. Phylogeny and metabolic potential of the methanotrophic lineage MO_3_ in Beijerinckiaceae from the paddy soil through Metagenome-Assembled Genome Reconstruction. Microorganisms. 2022; 10(5): 955.
